# Cryptococcose neuroméningée chez une patiente séronégative pour le VIH atteinte de tuberculose pulmonaire au service de Maladies infectieuses et tropicales du CHU du Point G de Bamako, Mali

**DOI:** 10.48327/mtsi.v2i4.2022.282

**Published:** 2022-10-19

**Authors:** Ouo-Ouo LOUA, Amavi Essénam ALLE AKAKPO, Dramane OUEDRAOGO, Yacouba CISSOKO, Mariam SOUMARÉ, Issa KONATÉ, Sounkalo DAO

**Affiliations:** 1 Service de Maladies infectieuses et tropicales du Centre hospitalier universitaire du Point G, Bamako, Mali; 2 Faculté de Médecine et d'odontostomatologie de l'Université des Sciences, des techniques et des technologies, Bamako, Mali; 3 Centre de recherche et de formation sur la Tuberculose et le VIH (CEREFO), Bamako, Mali

**Keywords:** Cryptococcose, Séronégative au VIH, Tuberculose, Hôpital, Bamako, Mali, Afrique subsaharienne, Cryptococcosis, HIV-negative, Tuberculosis, Hospital, Bamako, Mali, Sub-Saharan Africa

## Abstract

La cryptococcose neuroméningée et la tuberculose pulmonaire sont respectivement des infections mycosique et bactérienne graves survenant chez un sujet quel que soit son statut sérologique au VIH. Nous rapportons ici un cas de cryptococcose neuroméningée associée à une tuberculose pulmonaire et une dénutrition chez une patiente séronégative pour le VIH ayant un taux de CD4 à 750/mm^3^, pour mettre en exergue quelques rares particularités opposées à certaines littératures. Il s'agit d'une patiente de 18 ans, ménagère, originaire de Bamako, admise dans le service le 13 mars 2022 pour fièvre et altération de la conscience. Sa symptomatologie serait d'installation progressive en un mois, précédée de céphalée en casque rebelle au paracétamol, vomissements en jet et toux sèche, initialement traitée pour paludisme confirmé et suspicion de méningite. Chez qui il n'y a pas d'antécédent médico-chirurgical connu, ni usage de dermocorticoïde, ni de thérapie immunosuppressive et non alcoolo-tabagique. Les diagnostics de cryptococcose neuroméningée, tuberculose pulmonaire et dénutrition ont été retenus devant des arguments cliniques et microbiologiques. Aucune autre pathologie immunodépressive n'a été retrouvée. La patiente a été traitée avec succès par les antituberculeux oraux de première ligne et le fluconazole en perfusion. Trois intérêts sont tirés de ce cas clinique: la cryptococcose neuroméningée n'est pas que l'apanage du sujet VIH-positif, un taux élevé de CD4 ne signifie pas toujours une immunocompétence et le fluconazole est une alternative thérapeutique efficace contre la cryptococcose neuroméningée.

## Abstract (en)

Neuromeningeal cryptococcosis and pulmonary tuberculosis are respectively serious mycotic and bacterial infections occurring in a subject regardless of its HIV serological status. We report here a case of neuromeningeal cryptococcosis associated with pulmonary tuberculosis and malnutrition in an HIV-seronegative patient with a CD4 count of 750/mm^3^, to highlight some particularities opposed to certain literatures. This is an 18-year-old patient, housewife, from Bamako, admitted in the Infectious and tropical diseases department of the University teaching hospital Point G of Bamako on March 13, 2022 for fever and impaired consciousness. Her symptomatology appears to have gradually set in over a month, preceded by headache resistant to paracetamol, jet vomiting and irregular dry cough, initially treated with ceftriaxone, artesunate and paracetamol for confirmed malaria and suspicion of bacterial meningitis before admission. In whom no known medical-surgical history, no use of topical corticosteroids, no immunosuppressive therapy, no alcohol or tobacco, and no immunosuppressive pathology was found. The diagnoses of neuromeningeal cryptococcosis, pulmonary tuberculosis and undernutrition were retained in view of clinical and microbiological arguments. Diabetes, sickle cell disease, viral hepatitis B and C, kidney failure and cancer, which are immunosuppressive pathologies, were not found. She was successfully treated with first-line oral antituberculous drugs and fluconazole infusion. Three interests are drawn from this clinical case: neuromeningeal cryptococcosis is not only the prerogative of HIV-positive subjects, a high CD4 count does not always mean immunocompetence and fluconazole is an effective therapeutic alternative for neuromeningeal cryptococcosis.

## Introduction

La cryptococcose neuroméningée (CNM) est une mycose profonde sévère du système nerveux central, due au complexe *Cryptococcus neoformans* / *Cryptococcus gattii* [3, 6]. Rare chez l'immunocompétent, elle survient souvent au cours du SIDA lorsque le taux de CD4 est inférieur à 100 cellules/mm^3^ [3, 5, 13, 25]. Chaque année dans le monde, environ 957 900 cas de CNM surviennent, entraînant 624 700 décès, dont la majorité en Afrique subsaharienne. Au Mali elle semble être rare, mais d'une létalité de 62,5% [1, 13, 22]. La tuberculose, quant à elle, est une infection évolutive due au complexe *Mycobacterium tuberculosis,* survenant à tout stade de l'infection par le VIH [11, 12]. En 2020, l'OMS a recensé 9,9 millions de cas de tuberculose avec 1,5 million de décès dans le monde, affectant plus l'Afrique [20] et restant fortement endémique au Mali [24]. Ces infections peuvent aussi survenir chez des sujets VIH-négatifs, à l'occasion d'autres causes immunodépressives [7, 8, 28].

Nous rapportons ici un cas de CNM chez une patiente VIH-négative atteinte de tuberculose pulmonaire avec une malnutrition, pour mettre en exergue la possibilité de la survenue d'infection opportuniste (IO) chez un patient quels que soient son statut sérologique VIH et son taux de CD4.

## Cas Clinique

**Figure 1:**
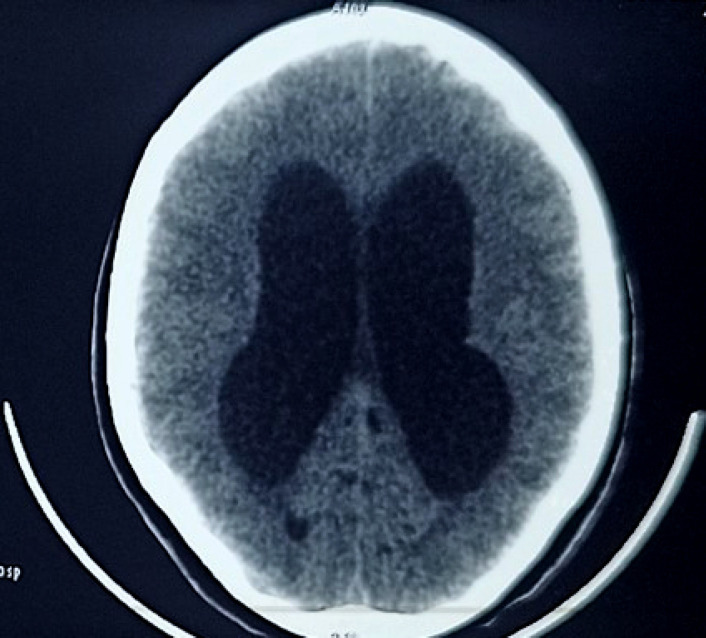
Tomodensitométrie cérébrale en coupe axiale avant injection de produit de contraste: dilatation tétraventriculaire associée à des hypodensités diffuses de la substance blanche chez une patiente de 18 ans séronégative pour le VIH, atteinte de cryptococcose neuroméningée et tuberculose pulmonaire Cerebral CT scan in axial section before injection of contrast medium: tetraventricular dilation associated with diffuse white matter hypodensity in an 18-year-old HIV-negative patient with neuromeningeal cryptococcosis and pulmonary tuberculosis

**Figure 2:**
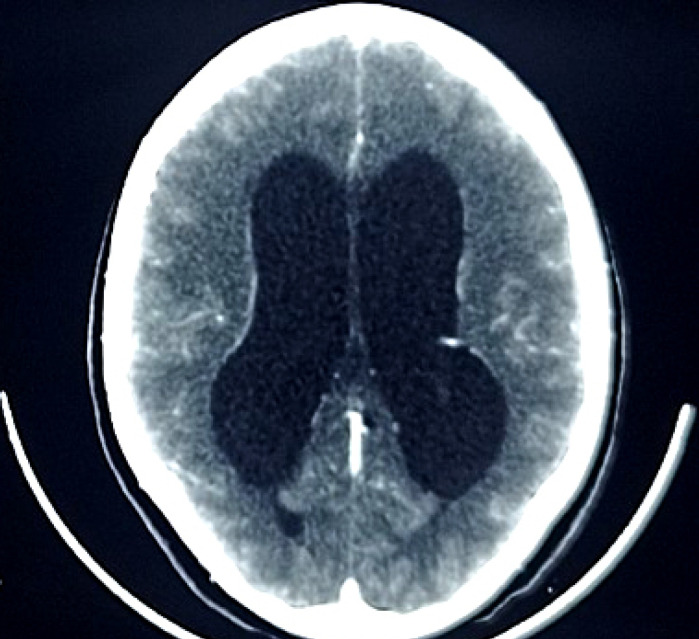
Tomodensitométrie cérébrale en coupe axiale après injection de produit de contraste: dilatation tétraventriculaire associée à des hypodensités rehaussées et diffuses de la substance blanche chez une patiente de 18 ans séronégative pour le VIH, atteinte de cryptococcose neuroméningée et tuberculose pulmonaire Cerebral CT scan in axial section after injection of contrast agent: tetraventricular dilation associated with enhanced and diffuse white matter hypodensity in an 18-year-old HIV-negative patient with neuromeningeal cryptococcosis and pulmonary tuberculosis

Patiente de 18 ans, ménagère, originaire de Bamako, admise dans le service le 13 mars 2022 pour fièvre permanente, toux sèche irrégulière, altération de la conscience précédée de céphalée en casque rebelle au paracétamol, et vomissement en jet. Sa symptomatologie serait d'installation progressive en un mois, initialement traitée dans un centre médical à base de ceftriaxone, artésunate et paracétamol pour paludisme confirmé et suspicion de méningite bactérienne, sans aucune thérapie immunosuppressive. Elle a une notion de contage tuberculeux 6 mois auparavant, sans antécédent médico-chirurgical, non alcoolo-tabagique, ni usage de dermocorticoïde. L'examen physique a objectivé une altération de l’état général, un score de Glasgow à 8/15, un syndrome méningé fébrile (38,6 °C), un poids à 33 kg et un IMC à 13,56 kg/m^2^. La tomodensitométrie cérébrale a mis en évidence une dilatation tétraventriculaire et des hypodensités diffuses de la substance blanche, rehaussées après injection du produit de contraste (Fig. 1 et Fig. 2). La radiographie du thorax est revenue normale. Dans le liquide de tubage gastrique (LTG), la bacilloscopie est revenue négative, le test Xpert-MTB/GeneXpert a faiblement détecté le *Mycobacterium tuberculosis* non résistant à la rifampicine. Le liquide cérébro-spinal (LCS) était clair avec une pléiocytose à 10 leucocytes/µl (polynucléaires 59% et lymphocytes 41%); hyperprotéinorachie (0,89 g/l); hypoglycorachie (2,34 mmol/l); normochlorachie (118,8 mmol/l); normolactatorachie (4,56 mmol/l); présence de levure encapsulée, sphérique et bourgeonnante au microscope optique après coloration à l'encre de Chine; à l'University Clinical Research Center (UCRC) de Bamako, la culture sur milieu de Sabouraud sans actidione à 37 °C révèle, après 3 semaines d'incubation, des colonies arrondies, brillantes au début blanchâtres puis ocres, le test à l'uréase positif sur le milieu urée-indole, l'assimilation de l'inositol, la phénol oxydase attestée par la coloration marron des colonies sur le milieu de Pal modifié, l'inositol assimilé et le glucose non fermenté, en faveur du *Cryptococcus neoformans.* Le génotypage et le sérotypage n'ont pas été réalisés. Son taux de CD4 était à 750 cellules/mm^3^. L'hémogramme a retrouvé une hyperleucocytose (20 100/µl), à prédominance neutrophile (13 789/µl). Une hypoprotidémie (47,61 g/l). À la recherche des maladies immunodépressives, trois sérologies VIH sont revenues négatives, deux glycémies à jeun normales à 0,97 et 0,92 g/l, l’électrophorèse de l'hémoglobine normale (Hb-A à 97,8%, Hb-A2 à 2,2%), les sérologies (AgHBs, Ac anti-HBc, VHC) sont revenues négatives, le bilan rénal normal (clairance de la créatininémie à 160,58 ml/mn, rapport azoturie/azotémie à 191,5) et certains marqueurs tumoraux sont revenus normaux (alpha-fœtoprotéine à 6,5 ng/ml, antigène tumoral-125 à 27 U/ml, antigène tumoral-15-3 à 18 U/ml, antigène carbohydrate-19-9 à 12 U/ml, antigène carcino-embryonnaire à 0,92 ng/ml), nous permettant d’écarter respectivement l'infection à VIH, le diabète, la drépanocytose, les hépatites virales B et C, l'insuffisance rénale et le cancer.

Les diagnostics de tuberculose pulmonaire (TBp) et CNM avec dénutrition ont donc été retenus. La patiente a alors été mise sous antituberculeux oraux de première ligne suivant le régime 2RHZE/4RH à raison de 2 comprimés/jour à jeun et fluconazole 1 400 mg/jour en trois perfusions (phase d'induction), 400 mg/jour per os pendant 8 semaines (phase de consolidation), puis 200 mg/jour per os (phase d'entretien) pour une durée d'un an. Trois ponctions lombaires (PL) soustractives (70 ml/PL) à intervalle de 72 h ont été réalisées.

L’évolution favorable à 14 jours du traitement est marquée par une conscience normale, l'apyrexie et la coloration à l'encre de Chine du LCS négative. Au deuxième mois, le test Xpert-MTB/GeneXpert sur le LTG est revenu négatif. À la fin de la phase de consolidation, la patiente avait un bon appétit et un poids à 35 kg, une bonne tolérance thérapeutique et la culture du LCS sur milieu de Sabouraud est revenue stérile. Son exeat a été fait le 17 juin 2022.

## Discussion

La CNM survient chez les PvVIH surtout lorsque le taux de CD4 est inférieur à 100 cellules/mm^3^ [3, 5, 13, 25], contrairement à la tuberculose qui survient chez tout sujet [11, 12].

Le *Cryptococcus neoformans,* espèce cosmopolite, affecte les immunodéprimés et les immunocompétents, et est majoritairement connu responsable des formes sévères de CNM [26, 29]. Quant à l'espèce *C. gattii,* ancienne variété du *C. neoformans,* c'est une espèce tropicale et subtropicale actuellement distincte [6, 29], qui affecte non seulement les immunocompétents mais aussi les immunodéprimés dans 2,4 à 30% des cas et est responsable de lésions pseudo-tumorales cérébrales et pulmonaires [9, 26]. La cryptococcose causée par *C. gattii* est significativement moins fréquente dans le monde (moins de 20%) que celle causée par *C. neoformans* (80%) [15]. Dans les littératures parcourues, la fréquence de l'infection par le *C. gattii* selon l'immunité des sujets n'est pas clairement décrite. Au Mali, l'incidence de la CNM a régressé au cours des deux dernières décennies, passant de 14 cas en 2004 à 0,08 cas/an [13, 16] du fait que le dépistage VIH et le traitement antirétroviral précoces des sujets soient devenus routiniers. Sa prévalence chez les PvVIH et les sujets non-VIH est respectivement de 5,1 et 0,6%, et le *C. neoformans* est jusque-là la principale espèce responsable [16].

Dans la littérature, en dehors de l'infection parle VIH, les thérapies immunosuppressives et certaines pathologies (maladies auto-immunes, hémopathies malignes, drépanocytose, syndrome de Wiskott-Aldrich, ataxie-télangiectasie, aplasie médullaire, granulomatose chronique familiale, asplénie, cancer, diabète, tuberculose évolutive, malnutrition) entraînent l'immunodépression [2, 7, 8, 18, 28], favorisant donc l'apparition des IO comme la CNM.

Notre patiente était indemne de l'infection à VIH, diabète, drépanocytose, hépatites virales B et C, insuffisance rénale, cancer, thérapie immunosuppressive, et avait un taux de CD4 à 750 cellules/mm^3^, mais était atteinte de TBp évolutive et de dénutrition. L'association CNM-tuberculose chez le VIH-négatif a aussi été rapportée en Afrique [10]. Ainsi, nous en déduisons que la TBp évolutive et la dénutrition seraient responsables de la survenue de la CNM chez notre patiente, sous réserve de l'implication d'autres pathologies responsables d'une dysfonction lymphocytaire [4, 8, 21] non diagnostiquées.

Les arguments diagnostiques de la CNM retrouvés ont aussi été décrits dans des cas similaires [7, 10, 14, 21] et la TBp paucisymptomatique ou minime diagnostiquée est rare, excepté chez l'enfant [27].

Dans notre contexte, avec l'indisponibilité de l'amphotéricine B, traitement de référence de la CNM à la phase d'induction [7, 17, 21, 23], notre patiente a bénéficié du fluconazole à forte dose, du fait de son efficacité prouvée [10, 13, 14]. Nous avons augmenté de 25% les doses de 6 h et 14 h du fluconazole pour majorer sa biodisponibilité, en raison de son interaction avec la rifampicine [19].

## Conclusion

L'intérêt de ce cas clinique est triple: la cryptococcose neuroméningée n'est pas uniquement l'apanage du sujet VIH-positif, un taux élevé de CD4 ne signifie pas toujours une immunocompétence et le fluconazole à forte dose est une alternative efficace contre la CNM.

## Consentement Éclairé

Notre patiente a donné son consentement éclairé pour la publication de son dossier médical sous anonymat.

## Liens D'intérêts

Les auteurs ne déclarent aucun lien d'intérêt.

## Contribution Des Auteurs

Ouo-Ouo Loua: conception du cas clinique, prise en charge de la patiente, revue de littérature, rédaction du manuscrit.

Amavi Essénam Alle Akakpo, Dramane Ouedraogo: prise en charge de la patiente, revue de la littérature, apport critique, approbation de la version finale à publier.

Yacouba Cissoko, Mariam Soumaré, Issa Konaté, Sounkalo Dao: prise en charge de la patiente, apport critique, correction du manuscrit et approbation de la version finale à publier.

## References

[1] Abassi M, Boulware DR, Rhein J (2015). Cryptococcal Meningitis: Diagnosis and Management Update. Curr Trop Med Rep.

[2] Afane Ze E, Guiedem E, Okomo Assoumou MC, Pefura Yone EW (2013). Impact dépressif de l'infection tuberculeuse sur les cellules immunitaires de défense. Health Sci Dis.

[3] Bandadi FZ, Raiss C, Moustachi A, Lyagoubi M, Aoufi S (2019). Quarante cas de cryptococcose neuroméningée diagnostiqués en 21 ans au laboratoire de parasitologie de l'hôpital Ibn Sina de Rabat [Forty cases of neuromeningeal cryptococcosis diagnosed at the Mycology-Parasitology Department of the Ibn Sina hospital in Rabat, over a 21-year period]. Pan Afr Med, J.

[4] Bretaudeau K, Eloy O, Richer A, Bruneel F, Scott-Algara D, Lortholary O, Pico F (2006). Cryptococcose neuroméningée chez un sujet en apparence immunocompétent [Cryptococcal meningo-encephalitis in an apparently immunocompetent patient]. Rev Neurol (Paris).

[5] Chadli S, Aghrouch M, Taqarort N, Malmoussi M, Ouagari Z, Moustaoui F, Bourouache M, Oulkheir S (2018). Cryptococcose neuroméningée chez des patients infectés par le VIH au Centre Hospitalier Régional d'Agadir (région Souss-Massa, Maroc) [Neuromeningeal cryptococcosis in patients infected with HIV at Agadir regional hospital, (Souss-Massa, Morocco)]. J Mycol Med.

[6] Cogliati M (2013). Global Molecular Epidemiology of *Cryptococcus* neoformans and Cryptococcus gattii: An Atlas of the Molecular Types. Scientifica (Cairo).

[7] Doumbia AK, Togo P, Coulibaly O, Dembele A, Kane B, Diakite AA (2021). Cryptococcose neuroméningée compliquée d'hydrocéphalie chez un enfant VIH négatif. Rev Mal Infect Microbiol.

[8] Dumas G, Bigé N, Lemiale V, Azoulay E (2018). Patients immunodéprimés, quel pathogène pour quel déficit immunitaire? (en dehors de l'infection à VIH). Méd Intens Réa.

[9] Franco-Paredes C, Womack T, Bohlmeyer T, Sellers B, Hays A, Patel K, Lizarazo J, Lockhart SR, Siddiqui W, Marr KA (2015). Management of *Cryptococcus gattii* meningoencephalitis. Lancet Infect Dis.

[10] Gbané-Koné M, Ouali B, Mègne E, Diomandé M, Coulibaly AK, Eti E, Kouakou NM (2015). Cryptococcose neuroméningée et tuberculose osseuse chez un immunocompétent: un cas [Cryptococcal meningitis and bone tuberculosis in an immunocompetent: a case]. Pan Afr Med, J.

[11] Issa HH, Cissoko Y, Soumaré M, Veltomtoh LD, Loua OO, Ibrahim A, Keita A, Mao C, Coulibaly B, Ouattara K, Konaté I, Magassouba O, Sogoba D, Fofana A, Dembelé J, Kouyaté F, Kaboré M, Dao S (2021). Aspergillose pulmonaire concomitante à une tuberculose et une immunodépression au, VIH, à propos d'un cas au service de Maladies infectieuses du CHU du Point, G, Bamako-Mali. Jaccr Infectiol.

[12] Janah H, Souhi H, Kouismi H, Mark K, Zahraoui R, Benamor J, Soualhi M, Bourkadi JE (2014). Facteurs de risque de mortalité par tuberculose pulmonaire [Pulmonary tuberculosis mortality risk factors]. Pan Afr Med, J.

[13] Konaté I, Sissoko AS, Soumaré M, Dembélé JP, Cissoko Y, Tchana MF, Coulibaly B, Fofana A, Dramane S, Magassouba O, Maïga I, Dao S (2021). Aspects épidémio-cliniques et thérapeutiques de la cryptococcose neuroméningée au Départment de Maladies infectieuses et tropicales du Centre Hospitalier Universitaire du Point G. Rev Mali Infect Microbiol.

[14] Kouakou GA, Ello NF, Kassi NA, Keita M, Doumbia A, Mossou C, Kassi FK, Tanon A, Ehui E, Eholié SP (2017). Fluconazole 1200mg ou 800mg dans le traitement de la cryptococcose neuroméningée en Côte d'Ivoire [Fluconazole 1200mg or 800mg for cryptococcal meningitis treatment in Ivory Coast]. J Mycol Med.

[15] Meyer W, Gilgado F, Ngamskulrungroj P, Trilles L, Hagen F, Castaneda E, Boekhout T (2011). Molecular typing of the *Cryptococcus neoformans/Cryptococcus gattii* species complex. In Heitman J et al. (dir.), Cryptococcus: From human pathogen to model yeast.

[16] Minta DK, Dolo A, Dembele M, Kaya AS, Sidibe AT, Coulibaly I, Maiga II, Diallo M, Traore AM, Maiga MY, Doumbo OK, Traore HA, Pichard E, Chabasse D (2011). La cryptococcose neuro-méningée au Mali [Neuromeningeal cryptococcosis in Mali]. Med Trop (Mars).

[17] Molloy SF, Kanyama C, Heyderman RS, Loyse A, Kouanfack C, Chanda D, Mfinanga S, Temfack E, Lakhi S, Lesikari S, Chan AK, Stone N, Kalata N, Karunaharan N, Gaskell K, Peirse M, Ellis J, Chawinga C, Lontsi S, Ndong JG, Bright P, Lupiya D, Chen T, Bradley J, Adams J, van der Horst C, van Oosterhout JJ, Sini V, Mapoure YN, Mwaba P, Bicanic T, Lalloo DG, Wang D, Hosseinipour MC, Lortholary O, Jaffar S, Harrison TS, ACTA Trial Study Team (2018). Antifungal Combinations for Treatment of Cryptococcal Meningitis in Africa. N Engl J Med.

[18] Neyrolles O, Volatron AC (2008). Ce que l'on sait de la réponse immunitaire antituberculeuse chez l'homme. Rev Mal Respir.

[19] Nicolau DP, Crowe HM, Nightingale CH, Quintiliani R (1995). Rifampin-fluconazole interaction in critically ill patients. Ann Pharmacother.

[20] OMS (2021). Rapport sur la tuberculose dans le monde 2021. Organisation mondiale de la Santé.

[21] Pappas PG, Perfect JR, Cloud GA, Larsen RA, Pankey GA, Lancaster DJ, Henderson H, Kauffman CA, Haas DW, Saccente M, Hamill RJ, Holloway MS, Warren RM, Dismukes WE (2001). Cryptococcosis in human immunodeficiency virus-negative patients in the era of effective azole therapy. Clin Infect Dis.

[22] Park BJ, Wannemuehler KA, Marston BJ, Govender N, Pappas PG, Chiller TM (2009). Estimation of the current global burden of cryptococcal meningitis among persons living with HIV/AIDS. AIDS.

[23] Perfect JR, Dismukes WE, Dromer F, Goldman DL, Graybill JR, Hamill RJ, Harrison TS, Larsen RA, Lortholary O, Nguyen MH, Pappas PG, Powderly WG, Singh N, Sobel JD, Sorrell TC (2010). Clinical practice guidelines for the management of cryptococcal disease: 2010 update by the infectious diseases society of america. Clin Infect Dis.

[24] Sangho O, Ouattara S, Telly N, Ballayira Y, Coulibaly CA, Traoré B, DERSP (2021). Évaluation de la prise en charge des patients atteints de tuberculose pulmonaire pharmaco-sensible au Centre de santé de référence, Commune V de Bamako, 2015-2018. Rev Mal Infect Microbiol.

[25] Shirley RM, Baddley JW (2009). Cryptococcal lung disease. Curr Opin Pulm Med.

[26] Sloan DJ, Parris V (2014). Cryptococcal meningitis: epidemiology and therapeutic options. Clin Epidemiol.

[27] Turkova A, Wills GH, Wobudeya E, Chabala C, Palmer M, Kinikar A, Hissar S, Choo L, Musoke P, Mulenga V, Mave V, Joseph B, LeBeau K, Thomason MJ, Mboizi RB, Kapasa M, van der Zalm MM, Raichur P, Bhavani PK, McIlleron H, Demers AM, Aarnoutse R, Love-Koh J, Seddon JA, Welch SB, Graham SM, Hesseling AC, Gibb DM, Crook AM, SHINE Trial Team (2022). Shorter Treatment for Nonsevere Tuberculosis in African and Indian Children. N Engl J Med.

[28] Wachinou AP, Agodokpessi G, Agbodande A, Affolabi D, Esse M, Adjibode O, Anagonou S (2018). La tuberculose du sujet âgé en milieu africain: particularités épidémiologiques, diagnostiques et évolutives au Bénin [Tuberculosis in older persons in African setting: Epidemiological, diagnostic and evolutive features]. Rev Pneumol Clin.

[29] Xu J, Vilgalys R, Mitchell TG (2000). Multiple gene genealogies reveal recent dispersion and hybridization in the human pathogenic fungus *Cryptococcus neoformans*. Mol Ecol.

